# Three-Year Gait and Axial Outcomes of Bilateral STN and GPi Parkinson’s Disease Deep Brain Stimulation

**DOI:** 10.3389/fnhum.2020.00001

**Published:** 2020-02-11

**Authors:** Shanshan Mei, Robert S. Eisinger, Wei Hu, Takashi Tsuboi, Kelly D. Foote, Christopher J. Hass, Michael S. Okun, Piu Chan, Adolfo Ramirez-Zamora

**Affiliations:** ^1^Departments of Neurology and Neurosurgery, Norman Fixel Institute for Neurological Diseases, University of Florida, Gainesville, FL, United States; ^2^Department of Neurology, Xuanwu Hospital of Capital Medical University, Beijing, China; ^3^Department of Biomedical Engineering, University of Florida, Gainesville, FL, United States; ^4^College of Health and Human Performance, University of Florida, Gainesville, FL, United States

**Keywords:** deep brain stimulation, globus pallidus internus (GPi), subthalamic nucleus (STN), long-term effect, gait disability, axial symptoms, Parkinson’s diasese

## Abstract

**Objective**: To examine the short- and long-term clinical outcomes of the bilateral subthalamic nucleus (STN) and globus pallidus internus (GPi) deep brain stimulation (DBS) on gait and axial symptoms in Parkinson’s disease (PD) patients. Available data have been inconsistent and mostly short-term regarding the effect of both brain targets on gait and axial symptoms. We aimed to identify potential target specific differences at 3-year follow-up from a large single-center experience.

**Methods**: We retrospectively reviewed short-term (6-month follow-up) and long-term (36-month follow-up) changes in the Unified Parkinson’s Disease Rating Scale (UPDRS) Part II and III total scores of 72 PD patients (53 with bilateral STN-DBS and 19 with bilateral GPi-DBS). An interdisciplinary team made target-specific decisions for each DBS patient. We analyzed changes in gait and axial subscores derived from UPDRS II and III.

**Results**: In both the STN- and GPi-DBS cohorts, we observed no significant differences in gait and axial UPDRS derived subscores in the off-med/on stimulation state at long-term follow-up when compared to baseline. On-med axial scores remained similar in the short-term but worsened in both groups (STN, 2.23 ± 3.43, *p* < 0.001; GPi, 2.53 ± 2.37, *p* < 0.01) in the long-term possibly due to disease progression. At long-term follow-up, the UPDRS III off-med/on stimulation scores worsened but were persistently improved from baseline in both groups (−9.07 ± 13.9, *p* < 0.001).

**Conclusions**: The study showed that long-term both STN- and GPi-DBS had a similar effect on gait and axial symptoms in UPDRS derived subscores at 36-month follow-up despite potential baseline differences in criteria for selection of each target. More sophisticated measures of gait and balance beyond the categorical UPDRS score will be needed for future studies.

## Introduction

Debilitating and progressive axial features, including gait disturbances, postural instability, and postural abnormalities are frequently observed during Parkinson’s disease (PD) progression (Nutt et al., 2011). These symptoms have been associated with reduced mobility, loss of independence, and recurrent falls in some cases with subsequent injuries (Fasano et al., 2015). Collectively, the literature has suggested that at a decade or more after diagnosis, the axial symptoms predominate in motor performance and contribute to a disproportionate decline in the therapeutic response to standard dopaminergic treatment, although some symptoms can be improved with adequate doses of dopamine replacement therapy or physiotherapy (Krack et al., 2003; St. George et al., 2010; Castrioto et al., 2011; Eisinger et al., 2019). Although deep brain stimulation (DBS) is an established procedure for treating many of the motor symptoms and fluctuations in PD, the reports on the effects of neuromodulation on axial disability have been inconsistent and difficult to predict, particularly in the long term.

Axial motor symptoms can be improved in some patients, remain unchanged in others, or even worsen in a subset of patients after DBS (Xie et al., 2012; Pötter-Nerger and Volkmann, 2013; Collomb-Clerc and Welter, 2015; Di Giulio et al., 2019). Several factors can affect axial symptoms, including patient characteristics, DBS target, the precise positioning of the electrode within the nucleus and also the stimulation parameters (Tisch et al., 2007; Fasano et al., 2015; Ramirez-Zamora and Ostrem, 2018). The subthalamic nucleus (STN) and globus pallidus internus (GPi) are two common DBS targets utilized for the management of motor fluctuations in PD patients (Ramirez-Zamora and Ostrem, 2018). Previous studies have suggested that GPi-DBS might be associated with a milder long-term (more than 2 years) decline of axial signs, such as balance, freezing of gait (Ferraye et al., 2008), and postural instability (St. George et al., 2010; Fasano et al., 2015), while STN-DBS might provide greater improvement of axial motor symptoms in the short term (~1 year; St. George et al., 2010). Most of the DBS efficacy data from 1 to 2 years follow-up of STN-and GPi-DBS for PD were derived from controlled studies, however only a few data focused on the axial symptoms and gait impairment (Xie et al., 2012; Aviles-Olmos et al., 2014; Kim et al., 2019). The long-term efficacy of bilateral DBS (particularly GPi-DBS) has been less well established.

In this study, we conducted a retrospective analysis of a large data set of PD patients managed with DBS in order to determine the short-term and long-term outcome of axial symptoms and gait function following bilateral STN- and GPi-DBS performed in a single center.

## Materials and Methods

### Patients

The study was approved by the University of Florida (UF) Institutional Review Board (IRB). Informed consent was provided according to the IRB-approved UF INFORM protocol. The UF INFORM database is a widely-used large movement disorders database with demographic, clinical, and surgical data (Oyama et al., 2012). Patient information was anonymized and de-identified prior to analysis. Patients were eligible for enrollment if they had received a diagnosis of idiopathic PD from a movement disorders-trained neurologist and underwent bilateral DBS implantation surgery at the University of Florida from 2002 to 2015. The selection of target—either the GPi or STN—was reached by a standard of care interdisciplinary screening and discussion (Higuchi et al., 2016). Inclusion criteria were: (1) bilateral placement of DBS; (2) fulfill the UK PD Society Brain Bank Clinical diagnosis criteria (Hughes et al., 1992); and (3) patients must have received both on-med and off-med scores in the preoperative assessment, 6-month (considered between 3–9 months) and 36-month postoperative (considered between 33–39 months). Exclusion criteria were: (1) patients have more than two leads in one side or they do not have the same target on both sides; (2) patients experienced two operations on one side (revised or replaced the DBS lead).

### Surgical Procedure and Electrode Location

Preoperative imaging was used to determine possible stereotactic coordinates of the GPi or STN target before surgery for each specific patient. A safe trajectory was chosen by the neurosurgeon. The target nuclei were structurally identified by manually fitting a digitized and modified Schaltenbrand-Bailey atlas to each individual’s MRI through the identification of white and gray matter (Sudhyadhom et al., 2012). Microelectrode recordings and monopolar macro stimulation testing during surgery led to adjustments of the direct and indirect functional targets. All patients received Medtronic (Minneapolis, MN, USA) 3387 implants. The anatomical location of the DBS electrode was measured using a postoperative computed tomography (CT) scan. The measured electrodeposition was calculated and transformed into the normalized anterior commissure-posterior commissure (AC-PC) atlas space using the MRI and CT however the CT was obtained 4 weeks post-surgery to allow for edema and air to resolve (see Supplementary Materials). Neurostimulators were placed approximately 4 weeks later and activated during the first clinical visit for DBS programming. All surgeries were staged—that is, the first lead and second lead were implanted on different dates as this was the standard of care at the institution (see Table 1).

**Table 1:** Main baseline clinical characteristics of the patients with PD involved in the long-term study*.

	GPi (*n* = 19) Mean ± SD (Range)	STN (*n* = 53) Mean ± SD (Range)	*P*-value
Gender, M/F	13/6	39/14	0.666
Age of onset (years)	46.37 ± 7.00 (37–59)	48.61 ± 10.47 (30–66)	0.392
Age at surgery (years)	60.47 ± 7.61 (47–74)	58.55 ± 10.34 (35–76)	0.461
Duration between baseline score and first surgery (months)	4.98 ± 2.83 (0–11)	5.05 ± 3.18 (0–14)	0.892
Duration between first and second surgery (months)	12.63 ± 11.74 (5–47)	9.30 ± 10.68 (0–63)	0.260
Follow-up from baseline to the 36-month timepoint (months)	53.68 ± 11.37 (43–85)	50.21 ± 11.55 (32–97)	0.262
Duration of PD at baseline (years)	22.63 ± 6.69 (12–47)	20.98 ± 5.16 (9–35)	0.297
UPDRS-II**	19.53 ± 5.38 (10–31)	17.20 ± 6.35 (5–34)	0.160
UPDRS-III***			
Off-medication	42.79 ± 9.13 (28–68)	42.40 ± 13.91 (11–81)	0.909
On-medication	24.89 ± 11.86 (8–58)	24.91 ± 10.26 (9–53)	0.997
Dopaminergic responsiveness (%)	41.68 ± 22.04 (3.03–85.19)	39.01 ± 22.44 (−45.45 to 74.19)	0.823
Axial score dopaminergic responsiveness (%)	43.08 ± 30.98 (0–100)	46.97 ± 38.38 (−100 to 100)	0.783
Hoehn & Yahr			
Off-medication	2.89 ± 0.64 (2–4)	2.82 ± 0.90 (1.5–5)	0.260
On-medication	2.55 ± 0.62 (2–4)	2.30 ± 0.46 (1.5–4)	0.614
LEDD (mg)	1,238.11 ± 660.55	1,128.91 ± 402.03	0.505

*Plus-minus values are means ± SD. Baseline variables were compared between the two groups with the use of a two-group t-test, Mann–Whitney U-test, and chi-square test. **Scores on the Unified Parkinson’s Disease Rating Scale part II (UPDRS-II) range from 0 to 42, with higher scores indicating more severe disease. ***Scores on the Unified Parkinson’s Disease Rating Scale III (UPDRS-III) range from 0 to 108, with higher scores indicating more severe disease. GPi, globus pallidus internus; STN, subthalamic nucleus; SD, standard deviation; M, male; F, female; PD, Parkinson’s disease; UPDRS, Unified Parkinson’s Disease Rating Scale; LEDD, L-dopa equivalent daily dose.

### Assessments

The patient assessments were conducted before surgery (baseline) and then at 6 months (6 M) and 36 months (36 M) after surgery. The baseline information included age, gender, age of onset, and age at DBS implantation. The clinical assessment included the Unified Parkinson’s Disease Rating Scale (UPDRS) Part II and Part III. At baseline and during follow up, we obtained motor data from UPDRS-III in the on-medication and off-medication conditions with on-DBS in follow-up assessments. The off-medication condition was defined as being off dopaminergic medications for 12 h. The on-medication condition was defined as being the best statement after taking regular dopaminergic medications. We defined the gait score using UPDRS-II scores as the sum of the fall score (item 13), freezing of gait score (item 14), and walking score (item 15; Katz et al., 2015). We also defined an axial score using UPDRS-III scores corresponding to the sum of the stand from chair score (item 27), posture score (item 28), stability score (item 29), and postural stability score (item 30; Thevathasan et al., 2011; Bonenfant et al., 2017). the L-dopa equivalent daily dose (LEDD) was calculated using the method of Claire L et al (Tomlinson et al., 2010). Dopaminergic responsiveness was calculated with the following formula: [score (off-medication) − score (on-medication)] * 100/score (off-medication).

The primary analysis was the difference between the GPi group and the STN group in the mean change from baseline to 36 months in the gait and axial scores (off-med/on-stim and on-med/on-stim), UPDRS-II total scores, and UPDRS-III total scores (off-med/on-stim and on-med/on-stim). Additional *post hoc* analyses were conducted to assess the progression of symptoms between baseline and last follow-up at 36 months and between 6 months and 36 months in each target separately. DBS programming was by standard of care maximized by 6 months after surgery and we aimed to evaluate early differences in each target utilizing similar measures. In addition, we assessed the difference in the Hoehn & Yahr (H & Y) stage and UPDRS-III total score.

### Statistical Analysis

Univariate descriptive analyses were used for reporting sample-level demographic and clinical characteristics. The independent samples two-tailed *t*-test for normal distribution data or Mann–Whitney *U*-test for non-normal data were used to compare the age of onset, age at surgery, duration of follow-up, duration of PD, UPDRS-II score, UPDRS-III score, and Hoehn & Yahr between groups. A chi-square test was used to compare gender between groups. The Mann–Whitney *U*-test was used to compare the differences between groups in the mean change of gait score, axial score, UPDRS-II total score, and UPDRS-III total score from baseline to 36 months (Verschuur et al., 2019). The one-way repeated measures ANOVA was applied to test for an effect of DBS target and follow-up time for each score. A Bonferroni test was used for *post hoc* analyses. Two-sided *P*-values of < 0.05 were considered significant. The statistical analyses were performed using the IBM SPSS, version 23.

## Results

### Study Population

Seventy-two patients with complete data (19 with GPi-DBS and 53 with STN-DBS) were enrolled. Across all patients, the median time between the first and second surgery was 7 months (range, 0–62). The 36-month follow-up time point occurred at a mean (Mean ± SD) of 46.07 ± 11.19 and 35.39 ± 3.34 months after the first surgery and contralateral surgery, respectively. The main demographic and clinical characteristics are summarized in Table 1. No significant differences were observed in the baseline measures.

### Primary Outcome Measures

**Figure 1:**
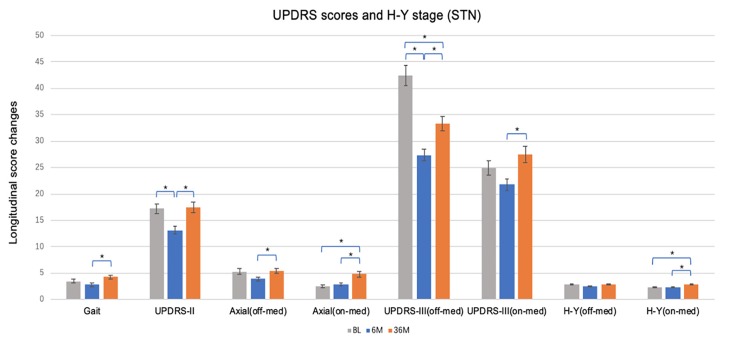
Short and long-term effects of bilateral brain stimulation of the subthalamic nucleus on gait score, Unified Parkinson’s Disease Rating Scale (UPDRS)-II, axial score, UPDRS-III and Hoehn & Yahr (H & Y) stage. STN, subthalamic nucleus; BL, baseline; 6 M, 6 months visit; 36 M, 36 months visit. Bars represent the mean and whiskers represent the standard error. *p < 0.05.

**Figure 2:**
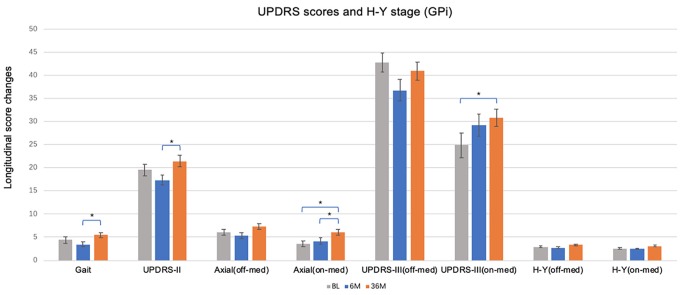
Short and long-term effects of bilateral brain stimulation of the globus pallidus internus on gait score, UPDRS-II, axial score, UPDRS-III and H & Y stage. GPi, globus pallidus internus; BL, baseline; 6 M, 6 months visit; 36 M, 3 months visit. Bars represent the mean and whiskers represent the standard error. *p < 0.05.

Table 2 shows the mean change from baseline to 6 months and baseline to 36 months in the STN (Figure 1) and GPi (Figure 2) groups for primary and secondary assessments. In both the STN- and GPi-DBS cohorts, we observed no differences in off-med/on stim axial and gait subscores at short-term or long-term follow-up compared to baseline. Whereas the on-med/on-stim axial score remained comparable between targets in the short-term, symptoms worsened (STN, *p* < 0.001; GPi, *p* < 0.01) at long-term follow up likely due to disease progression. Specifically, at 36 months compared to baseline the mean score of UPDRS II gait subscore increased by 1.11 ± 3.41 points in the GPi group and 0.80 ± 3.35 points in the STN group (STN vs. GPi, *p* = 0.642), the off-med/on-stim axial score worsened by 1.32 ± 2.85 in the GPi group and 0.09 ± 4.18 in the STN group (STN vs. GPi, *p* = 0.192), and the on-med/on-stim axial score worsened by 2.53 ± 2.37 (*p* < 0.01) in the GPi group and 2.23 ± 3.43 (*p* < 0.001) in the STN group (STN vs. GPi, *p* = 0.361).

**Table 2:** Mean change from baseline to 36-month in STN and GPi groups*.

	Mean score		Mean change from baseline to 6 M			Mean change from baseline to 36 M			Mean change from 6 M to 36 M		
	GPi	STN	GPi	STN	P**	GPi	STN	P**	GPi	STN	P**
**UPDRS-III Axial score (off-med/on-stim)**
Baseline	**6.00 ± 2.73**	**5.32 ± 3.52**
6 M	5.33 ± 2.66	3.88 ± 2.61	−0.61 ± 1.65	−1.46 ± 3.56	0.679						
36 M	7.32 ± 3.00	5.42 ± 3.57				1.32 ± 2.85	0.09 ± 4.18	0.192	2.00 ± 2.74	1.44 ± 2.44	0.423
**UPDRS-III Axial score (on-med/on-stim)**		
Baseline	**3.53 ± 2.80**	**2.57 ± 2.12**									
6 M	4.17 ± 2.70	2.86 ± 2.19	0.82 ± 1.62	0.36 ± 2.01	0.312						
36 M	6.05 ± 2.55	4.79 ± 3.77				2.53 ± 2.37	2.23 ± 3.43	0.361	1.82 ± 2.32	1.90 ± 2.71	0.805
**UPDRS-II Gait score**		
Baseline	4.37 ± 2.83	3.45 ± 2.50									
6 M	3.42 ± 2.19	2.78 ± 2.19	−0.95 ± 3.19	−0.67 ± 2.71	0.905						
36 M	5.47 ± 2.37	4.25 ± 2.82				1.11 ± 3.41	0.80 ± 3.35	0.642	2.05 ± 2.12	1.45 ± 2.14	0.302
**UPDRS-II Total**		
Baseline	19.53 ± 5.38	17.20 ± 6.35									
6 M	17.32 ± 4.60	13.16 ± 5.55	−2.21 ± 6.72	−4.04 ± 7.82	0.318						
36 M	21.42 ± 5.35	17.43 ± 6.77				1.89 ± 7.86	0.24 ± 7.73	0.592	4.11 ± 5.60	4.09 ± 5.70	0.928
**UPDRS-III Total (off-med/on-stim)**		
Baseline	**42.79 ± 9.13**	**42.40 ± 13.91**									
6 M	36.78 ± 9.92	27.36 ± 8.08	−6.06 ± 10.25	−14.96 ± 13.17	0.013						
36 M	40.95 ± 8.58	33.32 ± 10.05				−1.84 ± 10.26	−9.07 ± 13.95	0.045	4.22 ± 11.18	5.64 ± 8.15	0.597
**UPDRS-III Total (on-med/on-stim)**		
Baseline	24.89 ± 11.86	24.91 ± 10.26									
6 M	29.23 ± 9.90	21.78 ± 7.82	5.76 ± 8.24	−2.46 ± 10.30	0.002						
36 M	30.84 ± 8.23	27.40 ± 11.26				5.94 ± 10.98	2.49 ± 12.66	0.106	1.41 ± 8.94	5.46 ± 9.30	0.240
**UPDRS-III Total dopaminergic responsiveness (%)**						
Baseline	41.68 ± 22.04	39.01 ± 22.44									
6 M	19.06 ± 18.39	20.70 ± 21.41	−23.01 ± 27.17	−18.76 ± 26.17	0.531						
36 M	24.29 ± 13.76	19.08 ± 20.20				−17.39 ± 20.34	−19.93 ± 30.63	0.596	5.45 ± 18.70	−1.45 ± 28.63	0.378
**LEDD (mg)**						
Baseline	1,238.11 ± 660.55	1,128.91 ± 402.03									
6 M	1,333.42 ± 670.64	798.72 ± 478.04	95.32 ± 602.97	−330.19 ± 557.33	0.007						
36 M	1,463.47 ± 899.11	921.34 ± 533.61				225.37 ± 735.00	−207.57 ± 669.22	0.021	130.05 ± 406.92	122.62 ± 422.71	0.947

*Plus-minus values are means ± SD. **The P-values were calculated with the use of the Mann–Whitney U-test. GPi, globus pallidus internus; STN, subthalamic nucleus; M, month; UPDRS, Unified Parkinson’s Disease Rating Scale; LEDD, L-dopa equivalent daily dose; SD, standard deviation.

### Additional Analyses

At 6 months, STN patients experienced improvement in UPDRS II (−4.04 ± 7.82, *p* < 0.01) and III (−14.96 ± 13.17, *p* < 0.001) off-med/on-stim scores (Figure 1), with greater improvement in motor scores in both of the UPDRS III off-med and on-med scores compared to GPi patients (all *p* < 0.05). At long-term follow-up UPDRS III off-med/on-stim scores remained improved from baseline (−9.07 ± 13.9, *p* < 0.001) in patients managed with STN-DBS. The scores worsened compared to the initial benefit observed at 6 months (*p* < 0.001). In contrast, the UPDRS III on-med/on-stim scores did not significantly change at 6 months (−2.46 ± 10.30, *p* = 0.106) or 36 months (2.49 ± 12.66, *p* = 0.597) follow-up compared to baseline. Notable in this data, however, was the smaller sample size of the GPi and the potential differences in decision making favoring a GPi target.

When comparing change over time, there were no changes in UPDRS II or III off-med/on-stim during the duration of the study in patients treated with GPi-DBS (Figure 2). These observations may reflect the need for dopaminergic therapies for non-motor symptoms along with compensatory programming strategies to manage specific symptoms and to reduce adverse effects. Slight worsening in UPDRS III on-med/on stim score was noted in the GPi-DBS group as a whole at long-term follow-up (5.94 ± 10.98, *p* < 0.01). The off-med H&Y score did not change in the STN or GPi group at 6 months or 36 months compared to baseline, however, the on-med H&Y score did worsen at 36 months (0.53 ± 0.87, *p* < 0.001) in the STN group only.

## Discussion

We retrospectively assessed the short- and long-term changes in axial and gait symptoms in PD patients treated with bilateral GPi-DBS and STN-DBS. Our results revealed that at 36 months of follow-up, the effect of neuromodulation on axial and gait symptoms was comparable between the two targets. Previous studies reported that both GPi- and STN-DBS are effective in improving levodopa-responsive PD symptoms, however, effect on gait and axial symptoms have erratic and inconsistent responses to levodopa (Fasano et al., 2015). Nonetheless, in our cohort, only the on-med axial score worsened with follow-up.

To our knowledge, there are few reports primarily designed to address differences in axial and gait outcomes between bilateral GPi- and STN-DBS patients utilizing long-term data. Furthermore, prior published findings were derived from heterogeneous studies and populations. Rodriguez-Oroz et al. (2005) reported that there was a predominant deterioration of axial characteristics at 4 years follow-up, and the results were less striking for patients in the GPi group. The Netherlands SubThalamic and Pallidal Stimulation (NSTAPS) study showed that on some subscores GPi-DBS was less efficacious than STN-DBS in improving the axial symptoms at 1-year follow-up (Odekerken et al., 2013). Another randomized study showed (CSP study of 299 patients that GPi-DBS is superior to STN-DBS in improving gait symptoms in non-PIGD patients at 24-month follow-up (Katz et al., 2015). A recent meta-analysis of long-term results also revealed that a GPi group experienced improvement in the PIGD symptoms beyond 2 years, while the symptoms returned to preoperative levels in the STN-DBS group (St. George et al., 2010). Balance impairment, including falls, may more often occur after STN-DBS than GPi-DBS (Hariz et al., 2008). Although these reports have been inconclusive, it has been proposed that GPi-DBS might be a better target for severe gait difficulties (Celiker et al., 2019). Overall current data has been limited by a lack of objective gait assessments and clear separation among the different gait components and associated comorbidities.

These seemingly contradictory findings are not altogether surprising given that gait and axial symptoms are complex behaviors consisting of many sensorimotor subsystems that may not be fully characterized by the gait and axial items listed on the UPDRS. Although posture and gait are affected by bradykinesia, rigidity, and to a lesser extent tremor, other sensorimotor systems underlying posture and gait, such as dependent flexibility (Chong et al., 2000), sensory integration (Bronte-Stewart et al., 2002), and postural synergies (Horak et al., 2017), do not show the same responsiveness to levodopa. Therefore, each subsystem underlying control of posture and gait may be related to different neural circuits with varying sensitivities to levodopa or to DBS (Rocchi et al., 2002; Shivitz et al., 2006; Lyoo et al., 2007; Johnson et al., 2015). As STN and GPi project to different motor pathways within the CNS, stimulation at these sites may contribute differently to axial control (St. George et al., 2010). These different therapeutic outcomes of axial and trunk motor domains may reflect differential functional sub-loops of pathological motor network processing. This may be due to descending effects on the pedunculopontine nucleus or other non-dopaminergic centers in the mesencephalic locomotor area (Alam et al., 2011; Johnson et al., 2015).

In our cohort, the axial and gait scores increased at 36-month follow-up, especially in the on-med/on-stim axial score with a statistically significant change, and this differed from previous reports from other groups (Davis et al., 2006; Brosius et al., 2015; Collomb-Clerc and Welter, 2015; Kim et al., 2019). The gait score, which is composed of falls, freezing of gait and walking items, did not increase significantly at 36-month follow up. This is may have been due to the intrinsic limitations of our scales, lack of randomization, diaries and objective/specific markers of gait function, and assessment of freezing of gait and advanced disease. Disease duration was longer in our cohort compared with previous studies (22.63 ± 6.69 in the GPi group and 20.98 ± 5.16 in the STN group). In randomized controlled trials, for comparison the patients enrolled had a disease duration of approximately 10–12 years. Importantly, axial symptoms are known to be more prominent in patients with longer disease duration and severity. In the study by Rodriguez-Oroz et al. (2005), patients enrolled with 15 years of disease duration, 3–4 years after surgery, gait and postural scores worsened compared to baseline, especially in patients with STN-DBS. Other reports also noted increasing gait and balance difficulties at 2 years with STN-DBS (Celiker et al., 2019). Although DBS could consistently improve levodopa sensitive FOG (Brosius et al., 2015), there are cases of persistent or even worsening FOG after surgery (Davis et al., 2006; Collomb-Clerc and Welter, 2015).

The analysis of our secondary outcomes revealed differences between targets. Namely, the STN may be more effective than the GPi in reducing UPDRS-III total score, in the short-term, but in the long-term worsening of UPDRS scores appears to be less in patients treated with GPi-DBS. These findings are similar to previous reports (Holloway et al., 2010; Odekerken et al., 2016). Additionally, motor evaluation in the off state can be affected by the “long-duration response” of levodopa and its dose-dependent effect (Morgan and Sethi, 2005). Despite this finding might have been due in part to the use of higher doses of dopaminergic medications in the GPi-DBS group. The flexibility to use more dopamine and to adjust medications in the long-term may be an advantage to the GPi target, though this was not explored in the current study. There may be other factors, such as group size and predominant clinical features, that may account for these observed differences and not be controlled in this study (Rodriguez-Oroz et al., 2005).

Our study had several limitations. First, the analysis of the clinical data was retrospective. Second, a potential limitation of these data is that our samples were not well-matched, although baseline comparisons show these two groups to be similar, particularly with respect to our primary outcome (gait and axial scores). Moreover, the clinical features of all study subjects were systemically evaluated pre and postoperatively by well-trained neurologists after the same pre-operative evaluation protocol. STN- and GPi-DBS surgeries were performed by one neurosurgeon based on the same institutional guidelines. Notwithstanding these limitations, we found that PD patients treated with both GPi- and STN-DBS showed minimal change from baseline in gait and balance subscores and total UPDRS-II and UPDRS-III scores during the 36-month follow-up. A large problem with the data in this study was that the sample was not randomized and at our center more patients underwent bilateral STN DBS than GPi DBS. Future studies will be necessary to better delineate the relationship between lead location, axial scores. The use of objective gait markers and gait and falls monitors would greatly help to address many questions and also to document balance dysfunction which can impair outcomes despite a promising change in the UPDRS score. Finally, a study of sufficient sample size at a center performing bilateral STN and GPi DBS in a completely randomized fashion with long term follow-up of symptoms beyond the UPDRS scores will be necessary to be better understand the issues.

## Conclusion

In this cohort, we observed that both STN- and GPi-DBS had similar effects on gait and axial symptoms at long term follow up. Any benefits of STN-DBS in the short-term waned. Disease progression likely accounts for much of the axial and gait dysfunction however better metrics beyond the UPDRS for balance and other function will be necessary to understand long term functional impacts. In conclusion, GPi or STN are both viable DBS targets for the treatment of motor symptoms, and both cohorts will worsen over time in the UPDRS measured metrics of axial and gait symptoms.

## Data Availability Statement

The datasets generated for this study are available upon request to the corresponding author.

## Ethics Statement

The studies involving human participants were reviewed and approved by the University of Florida (UF) Institutional Review Board (IRB). The patients/participants provided their written informed consent to participate in this study.

## Author Contributions

SM, RE, and AR-Z were the major contributors in writing the manuscript. MO, KF, CH, and AR-Z contributed to the diagnosis and treatment of patients. WH, TT, MO, and PC contributed to checking the manuscript. All authors read and approved the final manuscript.

## Conflict of Interest

The authors declare that the research was conducted in the absence of any commercial or financial relationships that could be construed as a potential conflict of interest.
