# Supplementary material for: Three-Year Gait and Axial Outcomes of Bilateral STN and GPi Parkinson’s Disease Deep Brain Stimulation
Source: Front Hum Neurosci. 2020 Feb 11;14:1. doi: 10.3389/fnhum.2020.00001 (PMC7026192; doi:10.3389/fnhum.2020.00001)
Supplement: Supplementary file 1 [file Table_1.docx]

Average position of Contact 0

| Target | Absolute value of X (mm) (M±SE) | Y (mm) (M±SE) | Z (mm) (M±SE) |
| --- | --- | --- | --- |
| STN | 9.04±0.13 | -4.59±0.15 | -6.95±0.13 |
| GPi | 19.63±0.19 | -1.15±0.22 | -7.46±0.22 |

M = mean; SE = standard error; STN = subthalamic nucleus; GPi = globus pallidus internus; X = lateral-lateral position; Y = anterior-posterior position; Z = axial position; mm = millimeters

Normalized post-operative stereotactic coordinated for electrode 0 for both targets.
